# Expression patterns of heat-shock genes during stopover and the trade-off between refueling and stress response in a passerine migrant

**DOI:** 10.1007/s00360-023-01529-x

**Published:** 2024-01-31

**Authors:** Anastasios Bounas, Chrysoula Komini, Elisavet-Aspasia Toli, Artemis Talioura, Konstantinos Sotiropoulos, Christos Barboutis

**Affiliations:** 1 Department of Biological Applications and Technology, University of Ioannina, 45110 Ioannina, Greece; 2 Antikythira Bird Observatory, Hellenic Ornithological Society/BirdLife Greece, 52 Ag. Konstantinou Str., 10437 Athens, Greece

**Keywords:** BiP, Bird migration, Endoplasmic reticulum stress, Endurance exercise, Heat-shock proteins, qPCR

## Abstract

Migrating birds are often exposed to variable environments and face a multitude of stress exposures along their long-distance flights. During stopover refueling, migratory birds must balance the need to accumulate energy reserves to continue their migration with the need to respond to environmental and physiological stressors. We examined the gene expression patterns of different Heat Shock Proteins (HSPs) in migrating birds during stopover at different body condition states (lean vs. fat), to provide some first insights on the role of HSPs in bird migration and explore the concept of a trade-off between refueling and stress response. Our results showed upregulation of HSP expression at release that could be associated with muscle growth and increased cholesterol and lipid synthesis needed for birds to fuel their upcoming migration. On the other hand, during capture, upregulation of HSP5 could be attributed to physiological recovery from the non-stop endurance flight when crossing the Sahara Desert-Mediterranean Sea ecological barrier. All birds significantly increased their fuel loads up to 48% of lean body mass and we provide evidence for muscle rebuilding during stopover as flight muscle mass increased by 10%, highlighting the fact that stopover sites can play a major role in the physiological recovery of migrants.

## Introduction

When organisms are exposed to a variety of stress factors, they synthesis a group of highly conserved proteins called Heat Shock Proteins (HSPs), which are involved in “house-keeping” functions in the cell, acting as molecular chaperones. Their primary role is to assist in protein folding, prevent protein aggregation, and protect cells from damage caused by stress (Lindquist and Craig 1988; Feder and Hofmann 1999). HSPs are divided into several families according to their molecular weight, such as HSP90, HSP70, HSP60, HSP40 as well as small HSPs, and despite the fact that each family of HSPs has unique roles and cellular locations, they all work together to promote cell survival under stressful circumstances and maintain cellular homeostasis (Lindquist and Craig 1988; Sarkar and Roy 2017). To fully understand the cellular response to stress and how it affects diverse biological processes, it is essential to comprehend the roles and the regulation of heat-shock genes.

Natural populations are constantly exposed to environmental stressors as a result of changing environmental conditions. The HSPs operate as a buffer against this environmental variation and are crucial for maintaining homeostasis, thus directly influencing fitness of organisms, so their regulation is extremely important from an evolutionary and ecological point of view (Sørensen et al. 2003). Migrating birds are often exposed to variable environments and can face a multitude of occasional stress exposures along their long-distance flights (Lindstrom et al. 2014). Furthermore, migration is energetically expensive and migrant birds must often halt their journey at stopover habitats along the migratory route to rest and refuel (Wikelski et al. 2003). However, they often must also recover physiologically from the preceding migratory endurance flight, and cope with several stressors including muscle catabolism, organ recovery, hyperthermia, water stress, oxidative stress, and constitutive immune function (Guglielmo et al. 2001; Schmaljohann et al. 2022).

Even though migration is highly energy-demanding and stressful, HSPs expression in migrant birds has never been examined so far. HSPs could play a crucial role in cellular stress response and the maintenance of protein homeostasis whereas it has been suggested that upregulation of HSPs is not only part of the response to sudden extreme stress but also can have much wider ecological implications, as a response to less severe but regular stress exposure (Sørensen et al. 2003). One study examining natural variation in HSP gene expression in a songbird, has shown that populations show different neural HSP expression patterns according to their latitude thus providing a role for HSPs as a potential mechanism involved in thermotolerance (Woodruff et al. 2022). However, HSP expression can vary as a response to a wide range of stressful conditions other than temperature and can have significant implications for the recovery and survival of organisms (Lindquist 1986).

During stopover refueling, migratory birds must balance the need to accumulate energy reserves to continue their migration with the need to respond to environmental and physiological stressors. This balancing act can be challenging, especially for birds that arrive to stopovers energy-depleted (Bounas et al. 2023). The upregulation of HSPs itself can also be energetically costly, as it requires the synthesis and maintenance of additional proteins. In fact, HSP expression has been found to be rather costly in terms of fecundity, energy, growth, and survival (Sørensen et al. 2003). Such costs are believed to result from the suppression of normal cell processes during response to stress, the significant energy expenditure needed, and the toxic effects of high HSP concentrations caused by their interference with normal cell function (Feder and Hofmann 1999). We therefore argue that migratory birds may experience a trade-off between refueling and stress response.

Here, we examined the gene expression patterns of different HSPs in migrating birds during stopover at different body condition states (lean vs. fat), after the crossing of an extended ecological barrier. We aim to provide some first insights on the role of HSPs in bird migration, investigate the metabolic costs of migration and explore the concept of a trade-off between refueling and stress response. This will allow to shed light on how migratory birds manage to undertake such long and demanding journeys, and how they are able to cope with the many environmental stressors they encounter along the way.

## Materials and methods

### Sample collection and experimental setup

Eight migrating Garden warblers (*Sylvia borin*) were captured during spring migration of 2022 (April 21 to May 7) using 16 × 16 mm mesh, nylon, mist nets in Antikythira Island, an important migration stopover site (Barboutis et al. 2022), located in the Eastern Mediterranean (35° 51′ N, 23° 18′ E), within the standardized monitoring program of migratory birds run by the Hellenic Ornithological Society and the Hellenic Bird Ringing Centre. Birds were ringed, measured, and weighted to the nearest 0.1 g according to Svensson (1992) and then housed in individual cages in natural conditions for 5 days, as described in Bounas et al. (2023). In brief, we selected lean birds for collection to represent individuals that would use the island as a stopover to refuel and not resume their migration during the upcoming night, based on their body mass measurements. Mean arrival body mass of garden warblers caught on Antikythira Island during spring is estimated at 16 g (Barboutis et al. 2011), and therefore we kept lean birds (body mass between 14.0 and 15.2 g) in individual cages measuring 50 × 30 × 30 cm, built from nonmagnetic material (PVC and wood) while lined in all sides with white semi-transparent cotton fabric to provide natural light conditions. All individuals were provided with food (mealworms, *Tenebrio molitor*) and water ad libitum. Pectoral muscle shape was estimated for the first (at capture) and fifth (at release) day shortly before migratory birds were released using a muscle meter (Bauchinger et al. 2011), adopting the 3D-printable version designed by Powell et al. (2021). Three independent measurements were taken, and the average value was used for subsequent analyses. Blood samples (50 μl) were collected from each bird twice, also at capture and release, during morning hours (8:00–10:00 h), by puncturing a brachial wing vein with a 25-gauge needle. Blood was immediately stored in DNA/RNA Shield buffer (Zymo Research, Orange, CA, USA) that was initially kept at room temperature until shipment (within less than a month from collection) and then preserved at − 20 °C until RNA extraction, following Komini and Bounas (2022).

### RNA Isolation and quantification of HSP gene expression (qRT-PCR)

The Quick RNA Whole-Blood Kit (Zymo Research, Orange, CA, USA) was used for RNA purification following the manufacturer’s guidelines. RNA concentration, purity, and integrity were evaluated using standard spectrophotometric measurements whereas overall quality was measured through an Agilent 2100 Bioanalyzer RNA assay (Agilent Technologies, Santa Clara, CA, USA). Sample 260/280 ratio ranged from 1.7–1.97 (mean = 1.87) and RIN values ranged from 6.4–7.7 (mean = 7). We amplified five target genes encoding HSPs from three different families: DNAJA4 and DNAJB6 (Hsp40 family), HSPA2 and HSPA5 (Hsp70 family), and HSP90AA1 (Hsp90 family). Primers used to amplify the selected target genes were designed in Xie et al. (2014) and in Wang et al. (2021). One-step RT-qPCR was performed to determine HSPs expression levels at different body condition states, using the Kapa SYBR FAST One-Step Universal RT-qPCR kit (Kapa Biosystems) following the manufacturer’s instructions. GAPDH was used as a reference gene after Olias et al. (2014). The reactions were performed in a CFX Connect™ Optics Module thermocycler (BioRad) applying the appropriate thermal protocol: 42 °C for 5 min, 95 °C for 4 min and then 36 cycles at 95 °C for 2 s followed by 59 °C for 20 s. Each qPCR reaction for every sample was replicated twice. The amplification efficiency of each heat-shock protein gene was recorded, and their relative gene expression ratio was calculated using the 2^−ΔCT^ method. The intra-assay variation was 0.56%, while the inter-assay variation among three assays was 2.8%. Such CV values were considered acceptable to validate the repeatability and reproducibility of the assay.

### Data analysis

To estimate the fuel load (F), i.e., the bird’s body components which can be used as fuel, the following equation was used:

$$F{ = }\left( {m \, - \, m_{0} } \right)/m_{0}$$

where m, is an individual’s body mass and m_0_ is the birds’ body mass without any fuel. Fuel load was calculated for every bird at both body condition states (lean vs. fat), thus corresponding to the arrival fuel load (*F*_*a*_) and departure fuel load (*F*_*d*_) using the body mass value on day 1 and body mass value at day 5 respectively. To estimate body mass without fuel (*m*_0_), i.e., the body mass of a bird with both a fat score and a muscle score of 0, according to the structural mass concept (Salewski et al. 2009), we used the species-specific values provided from Antikythira stopover site (Barboutis et al. 2022) and used each individual’s wing chord measurement as input in the equation:

$$m_{0} = { 6}.{89 } + \, 0.0{8 } \times {\text{ wing length}}$$

To estimate total flight muscle mass at capture and at release we used muscle shape values from first and last day, body mass (m) from first and last day, and tarsus length of each individual as inputs to the equation for Garden Warblers developed by Bauchinger et al. (2011):

$${\text{Flight muscle mass }} = \, - {1}.{212 } + \, \left( {0.{293 } \times {\text{ muscle shape}}} \right) \, + \, \left( {0.0{45 } \times \, m} \right) \, + \, \left( {0.{199 } \times {\text{ tarsus length}}} \right)$$

All differences between body condition states states were compared by paired t tests and considered significant at *p* < 0.05. Values are reported as mean ± SE. Data were analyzed using R (R Core Team 2023).

## Results and discussion

We aimed to understand how migratory birds regulate HSP expression thus providing some insights on how they cope with the various stressors they encounter during migration. One possible scenario is that during stopover, migratory birds may upregulate the expression of certain types of HSPs to cope with the increased demand for protein folding and other stress-induced cellular processes. This could help to maintain cellular homeostasis and prevent protein misfolding or aggregation, which can lead to cellular damage and even cell death (Craig et al. 1994). It is also plausible that if birds are energy-depleted and need to recover before refueling, the expression of HSP genes may be upregulated to mitigate stressors such as food deprivation, decrease in body mass and muscle catabolism due to prolonged endurance flight, thus protecting cells from damage and facilitate recovery.

**Fig. 1:**
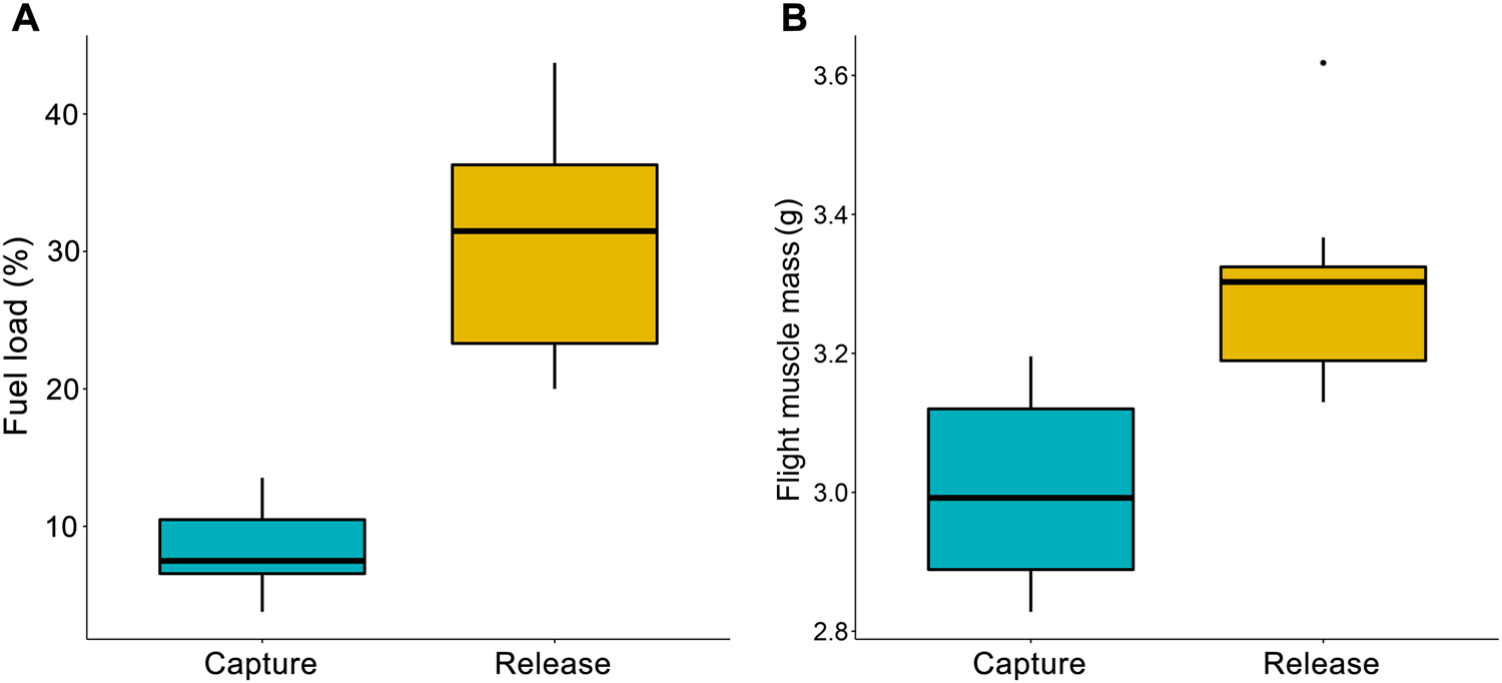
Boxplots showing Fuel load (A) and Flight muscle mass (B) measurements of individuals belonging in the two study groups (capture vs. release)

**Fig. 2:**
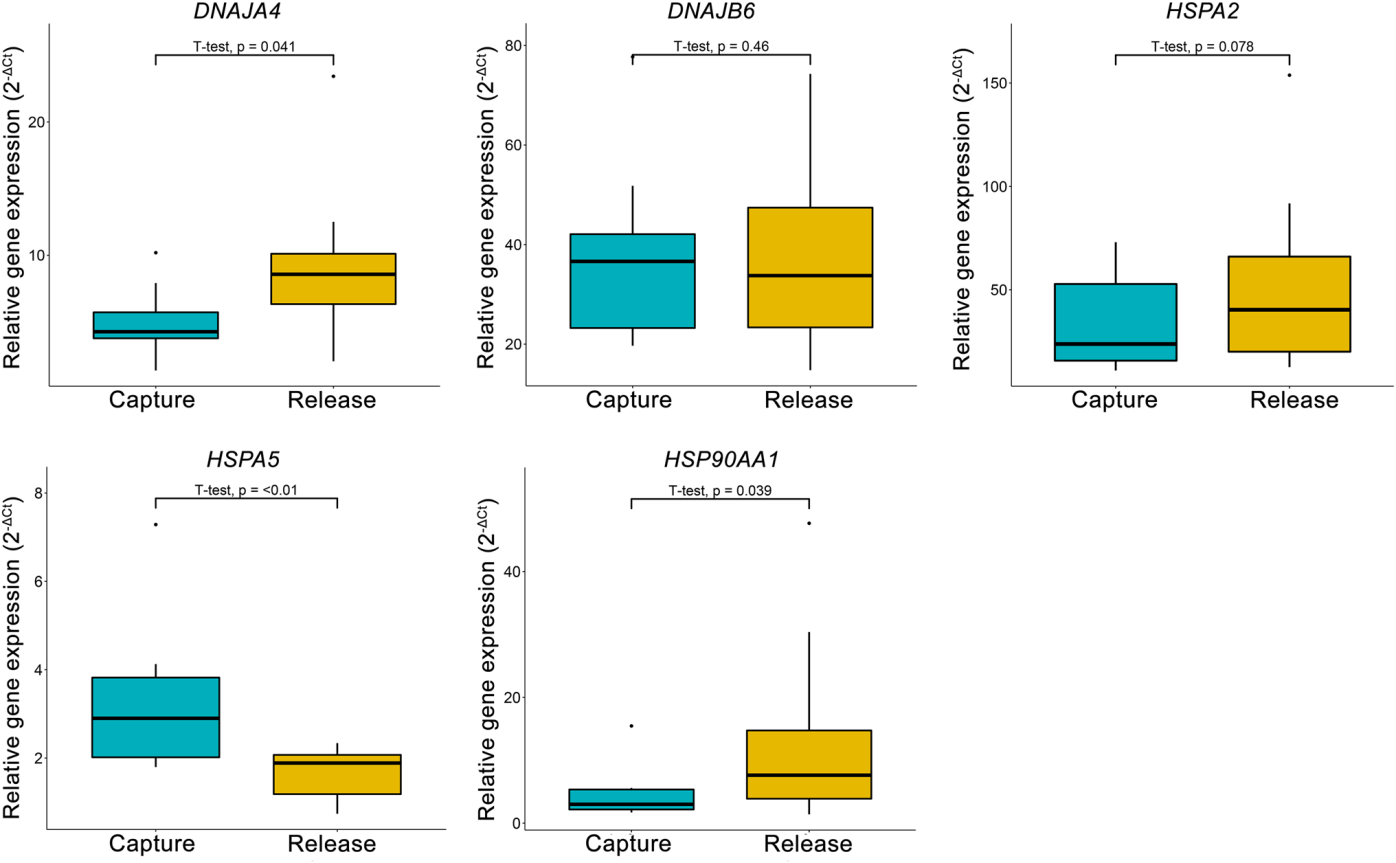
Relative gene expression values of individuals belonging in the two study groups (capture vs. release), measured for five heat-shock genes. *p* value of a paired t test between states is given for all comparisons

At the beginning of the experiment all birds showed signs of depletion, with their body mass ranging from 14–15.2 g (mean = 14.5 g) and fuel loads ranging between 3%-13% (mean *F = *8%). Furthermore, flight muscle mass ranged between 2.8–3.2 g (mean = 3 g). Our results show that all birds improved their condition by day 5 (Fig. 1), as fuel loads significantly increased, reaching 20–48% of lean body mass (*t = *6.81, *p* < 0.001). There was also evidence for muscle rebuilding as flight muscle mass increased by 10% (*t = *7.13, *p* < 0.001). Those observations are in line with the existing evidence that stopover sites can play a major role in the physiological recovery of migrants (Ferretti et al. 2021; Barboutis et al. 2022; Schmaljohann et al. 2022; Bounas et al. 2023). Out of the HSP genes examined, HSPA2 and DNAJB6 were not found to show any expression differences between body condition states, although HSPA2 showed a 1.6-fold change but was marginally insignificant (*p* = 0.07). DNAJA4 and HSP90AA1 levels increased 2.6 ± 0.3-fold and 4.5 ± 0.6-fold, respectively at release, whereas for HSPA5 the opposite pattern was observed, with elevated levels during capture (0.6 ± 0.07-fold lower at release; Fig. 2).

Specifically, the significant change in DNAJA4 at release (*t = *2.03, *p* = 0.04) could be associated with increased plasma cholesterol during the fattening period as shown for our study species (Totzke and Bairlein 1998). DNAJA4 is a chaperone protein involved in the cholesterol biosynthesis pathway (Robichon et al. 2006), thus increased cholesterol synthesis could play a major role in optimal digestion, absorption and accumulation of dietary fats and further improve the ability to gain weight and build muscle (Bairlein 2002, but see Araújo et al. 2019). In fact, increased DNAJA4 expression levels after feeding were observed in other organisms such as the Atlantic salmon and was associated with muscle growth (Bower and Johnston 2010). Similarly, HSP90AA1 upregulation at release may not only be related to lipid metabolism and adipogenesis that is needed for birds to fuel their upcoming migration but also on muscle physiology by regulating the transforming growth factor β (TGF‐β; Bazile et al. 2020). Any synergistic action of both those genes with other genes involved in response to endoplasmic reticulum (ER) stress and proposed to play a role in migration preparation (e.g., INHBE; Frias-Soler et al. 2022), remain to be examined.

The upregulation of HSPA5 in lean birds provides the most interesting case of response to the physiological challenges inherent to migration.. HSPA5 is a master regulator of ER homeostasis (Wang et al. 2017), so when homeostasis is disrupted, HSPA5 triggers the unfolded protein response (UPR) pathway to deal with the stress (Lee 2005; Schröder and Kaufman 2005). Several stressful conditions are experienced by birds arriving at the stopover site after crossing the Sahara Desert-Mediterranean Sea ecological barrier, such as oxidative stress and nutritional deprivation along with reduced digestive capacity as well as muscle fatigue due to the non-stop endurance flight (McWilliams et al. 2004; Skrip et al. 2015; Bohnert et al. 2018). All of the above conditions require proper regulation of ER stress and UPR^ER^ pathways (Yap et al. 2021). Therefore, increased concentrations of HSPA5 in blood may reflect an individual’s response to migration-induced stress and could be further examined as a potential biomarker for overall migration condition.

## Data Availability

The dataset generated during the current study is available from the corresponding author on reasonable request.
